# The feasibility of local participation in Measuring, Reporting and Verification (PMRV) for REDD+

**DOI:** 10.1371/journal.pone.0176897

**Published:** 2017-05-11

**Authors:** Manuel Boissière, Martin Herold, Stibniati Atmadja, Douglas Sheil

**Affiliations:** 1 Centre de Coopération Internationale en Recherche Agronomique pour le Développement (CIRAD), Montpellier, France; 2 Center for International Forestry Research (CIFOR)—Ethiopia, Addis Ababa, Ethiopia; 3 Center of Geo-Information, Department of Environmental Science, Wageningen University, Wageningen, The Netherlands; 4 Department of Ecology and Natural Resource Management, Norwegian University of Life Sciences, NO-1432 Ås, Norway; Pacific Northwest National Laboratory, UNITED STATES

## Abstract

The studies in this PLOS ONE collection investigated the feasibility of community participation in Measuring, Reporting and Verifying (Participatory MRV–PMRV) initiatives in the context of national programs to reduce emissions from deforestation and forest degradation (REDD+). While such participation is desirable, its feasibility has been uncertain. This collection builds the empirical foundations for putting PMRV into practice. The authors of this article identified five crucial considerations: (1) clarify the stakeholders, (2) understand their motivation to participate, (3) integrate knowledge and information from multiple disciplines and sources, (4) convey knowledge and information across multiple levels of governance, and (5) clarify and enable the links to REDD+ safeguards. We conclude that local communities and other local actors can play a major role in achieving REDD+ MRV, however, this requires attention to their needs and motivations. Future activities should include assessment of past PMRV experiences, costs and benefits, operationalization of reporting and verification, formalization of PMRV and full scale testing on the ground.

## Introduction

Mitigating the effects of climate change is a major global challenge. In November 2015, the 21^st^ Conference of the Parties (COP 21) of the United Nations Framework Convention on Climate Change (UNFCCC) in Paris, agreed to limit global temperature rise to less than 2°C compared to preindustrial levels ([1], Article 2:1(a) [2]); reduce emissions from deforestation and forest degradation [3], and conserve forests, manage forests sustainably and enhance forest carbon stocks (REDD+).

Measurement, Reporting and Verification (MRV) is a set of methods to monitor REDD+ activities and outcomes. MRV activities include measuring carbon stocks and the emissions of carbon dioxide and other greenhouse gases, reporting these measurements to higher (regional, national and global) levels, and verifying that estimations and reporting have been conducted correctly [4]. Apart from major carbon stores, forests are also home to many people whose lives are affected by deforestation. The interests of local communities appear to be poorly reflected in the internationally-driven REDD+ processes ([5], [6]), and it remains unclear how local people can contribute to and benefit from MRV.

REDD+ is increasingly not only about emissions reductions, it also includes social safeguards, livelihood benefits, biological conservation, and sustainable landscape development [1]. As an essential component of REDD+, MRV should monitor all these aspects. The UNFCCC [7] encourages local participation as part of REDD+ implementation and monitoring so that community members and other local stakeholders become empowered participants in the process rather than mere bystanders. Countries are encouraged to include social and environmental safeguards, clarify benefit sharing among the different stakeholders involved in REDD+ [5], and propose alternative livelihoods to prevent further deforestation and forest degradation. One of the ethical underpinnings of REDD+ is ‘to avoid doing harm’ to local communities [8] [9]. These principles also apply to MRV and PMRV.

Given the potential value of community participation in MRV for REDD+, how can it operate in practice? Past case studies have focused on tree measurement and biomass estimations (see [10] this collection, henceforth indicated by *, [11], [12], [13], [14]), leaving the other aspects of PMRV largely unexplored ([10], [15], [16], [17], [18]). How feasible is PMRV for the whole range of REDD+ concerns (e.g. carbon sequestration, drivers of forest cover change, social and environmental safeguards) [19]? In this collection we address this vital question through two review papers, and nine empirical studies based on research in Indonesia, Ethiopia, Mexico and China.

**Fig 1:**
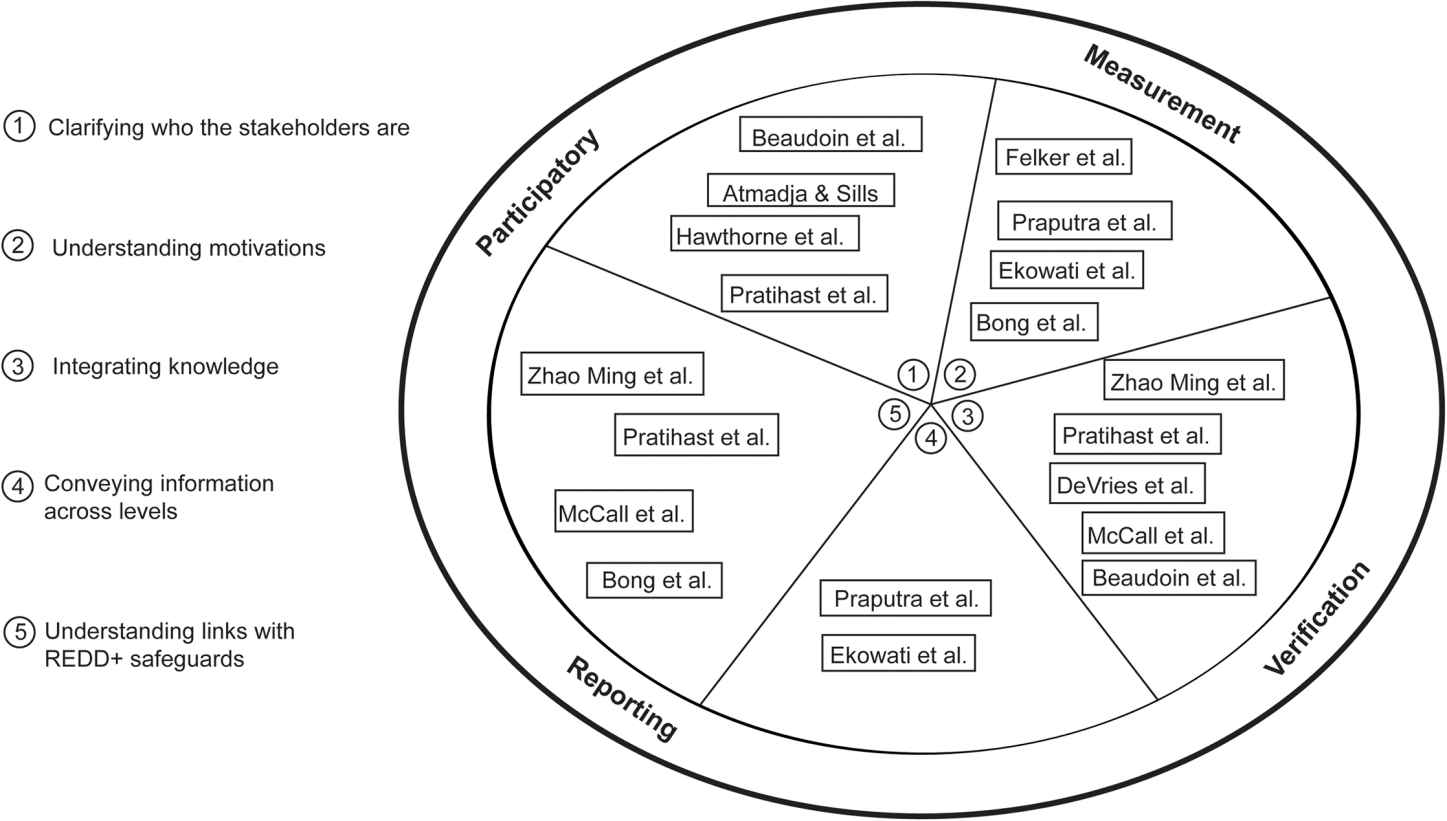
The papers in this collection and their relationship to the five crucial considerations.

This introductory article synthesizes the general lessons from the collection at the time of publication. We highlight five crucial considerations for ensuring PMRV is feasible (see Fig 1) and then examine some broader lessons and priorities for future research. The five considerations include the need to: (i) clarify who the **stakeholders** are, (2) understand stakeholders’ **motivation** to engage in MRV, (3) **integrate knowledge** from multiple disciplines and actors into MRV activities, (4) convey MRV information across **multiple levels of governance**, and (5) understand the links between PMRV and **REDD+ safeguards.**

## Five crucial considerations for PMRV

### 1. Clarify the stakeholders

PMRV depends on the collaboration of local communities, NGOs, local governments, the scientific and the international community.

#### Meaningful involvement of local people

The role of local people is currently limited to trainees or resource persons for collecting data on land use changes. Their participation needs to be integrated at higher levels, including the design of PMRV activities, and they need feedback from the national level on how decisions about forest management will impact them. Risks for local livelihoods (e.g. important economic activities being forbidden because of their environmental impact, see Bong et al [20]*) also need recognition.

Information based on community perceptions of forest cover changes, and indicators of social and environmental safeguards can ensure that MRV systems monitor beyond emission reductions. However, communities are heterogeneous, with people having different views and aspirations. There are practical limits to generalizing data and describing the population as a whole. Such limits are often unclear in reports that often describe inadequately how the data was collected and aggregated (see [21]*). PMRV activities that plan to make use of community members’ perceptions need to be mindful of the methods by which the perceptions were elicited from individuals, and used on behalf of a larger community.

What are the benefits and caveats of engaging local communities in MRV? Analyzing the literature on PMRV, Hawthorne et al. [10] found several claims to how PMRV can benefit REDD+ programs, especially with regard to producing accurate, low-cost carbon measurements. In contrast, there is little evidence of negative effects of PMRV with regard to equity in benefit sharing and inaccurate reporting by local people if financial rewards are linked to performance. PMRV claims have seldom been based on empirical findings [10]. This may be due to the limited number of empirical PMRV studies, each with its own limitations, and few opportunities to test how PMRV affects existing REDD+ components.

#### Involving other stakeholders

PMRV requires more than local people. Setting up the program, training, supervision, trouble shooting, quality control and data interpretation will all require a certain expertise. Local NGOs working with local people can facilitate and also relay reported data collected by/with local people to the national level ([22]*). The government should aggregate data from subnational sites, integrate the data into the national database, validate the data collected and report the outcomes to the international level.

### 2. Understanding stakeholders motivations to participate

Understanding the motivations and incentives of local people to participate in an affordable and effective PMRV system is important to create a local sense of ownership and ensure sustainability of PMRV, with local people available and interested in taking part in long-term activities. Ekowati et al [23]* explored the case of the Indonesian child health monitoring program, centered around the *Posyandu* (village healthcare posts). This PMRV system has existed for decades, reaching the most remote, rural parts of Indonesia and offering only minor financial incentives to the (mostly women) collaborators. The authors found that the main motivations for participating were (1) personal interest in the work, (2) belief that their participation benefits the community, and (3) engagement by respected persons. While collaborators receive small payments, they are incentivized by other, non-financial, benefits, such as recognition by their community and others within the health system, and by the opportunities they gain to participate in training programs.

Also Felker et al. [24]* found that non-financial incentives were important for carbon monitoring (e.g. for tree measurements), especially incentives in the form of increased legal recognition of land rights. However, such incentives are related to local land tenure arrangements, which differ across their study sites. For example, different parts of a village forest are generally under various authorities, based on either statutory or customary claims. These various authorities could guide PMRV actors and activities.

Recognition of customary lands and associated rights may encourage communities to participate in REDD+ activities. Often tenure and boundaries of these lands are unclear and contested. Beaudoin et al. [25]* found that participatory mapping can provide a forum for discussion between villagers and government authorities to clarify village and individual land boundaries. These discussions can help identify and solve possible conflict over land use and land tenure between neighboring villages and/or with the government.

Perceived land tenure security/insecurity is probably more important in this regard than legal tenure security. For example, people in the Java study sites do not feel secure with land certificates, which are arguably the most legally secure land documents available [24]. Some have agreements with Perhutani (i.e., the Government owned Forestry Enterprise), which grants them limited access and user rights to the tree plantations. They feel insecure because they do not know how long the agreement(s) will last and what will happen next. In contrast, people in Papua who do not have any land certificates, have a strong sense of ownership and trust in customary land tenure that–according to the villagers interviewed–surpasses statutory tenure [25, 26].

PMRV activities in a village forest would need first to secure authorization from the village head, representing the statutory authority. However, a village forest (or parts of) can traditionally belong to groups or clans under the authority of a traditional leader (e.g. in Papua), and so securing authorization from these traditional leaders would be an essential second step. The traditional leaders could also provide guidance in selecting villagers to help with the activities.

When discussing the potential benefits from PMRV activities, the heterogeneous nature of communities must be considered. For example, some villagers might prefer payments to benefit the entire community, while others may prefer individuals to receive the benefits, i.e. those directly involved in the PMRV activities or having customary rights to the land where activities are conducted. Bong et al. [20] suggest benefit sharing is judged on how much local livelihood activities are impacted by REDD+ with those suffering greater costs receiving greater compensation. Benefit sharing arrangements should take into account the potential conflicts that may arise when different user groups within a village lay claim to land, resources and forest-based livelihoods.

### 3. Integrating knowledge and information from multiple disciplines and sources

Participatory MRV requires the collection of data at the local level and the use and integration of different sources of information. In some cases, using a combination of modern technology and local knowledge can benefit MRV. The use of GPS and smartphones, as well as biomass assessments, data analysis and reports writing skills can also contribute to MRV. Although local people know the land use history of their territory and the drivers of deforestation and forest degradation, this knowledge will still need to be standardized for it to be integrated into the national MRV system and databases.

Data collection can be standardized as part of an integrative and interactive system that builds communication and sharing among the different stakeholders (e.g. villagers, government MRV agencies, civil society). Pratihast et al. [27]* describe the design and implementation of such a (web-based) system to monitor the UNESCO Kafa Biosphere Reserve in Southwestern Ethiopia. Information collected through this web-based system was consistent over time and aggregated in a standardized way. Their system supports PMRV because it is interactive, dependent on local monitoring data, and provides feedback to local people, something Ekowati et al. highlighted as vital for PMRV [23].

DeVries et al. [28]* give another example of knowledge integration from Ethiopia. They combined a stream of data from smart phones used by local people with Landsat time series to characterize and map deforestation and forest degradation. The locally recorded forest-changes offer deeper understanding of forest change processes recorded by satellites, especially the changes below the canopy.

Bong et al. [20] emphasize the importance of local knowledge of deforestation and forest degradation dynamics at the local level. Integrating this local knowledge is vital for MRV success because it allows for site-specific assessment and monitoring of drivers of land use change. Vega Praputra et al. [22] suggest using simple data formats, based on existing forest reporting systems, which are agreed by all stakeholders. This should include a clear description of community reporting responsibilities and benefits.

Beaudoin et al. [25] stress that MRV costs could be minimized by limiting the sampling area for ground-truthing to areas where remote sensing and participatory mapping data are inconsistent. This can save time and may trigger local interest and participation in ground checks (i.e. verification) and carbon measurements. Participatory mapping can provide data on social contexts (e.g., tenure status, historical events) and land use dynamics that are either impossible or difficult to accurately obtain from remote sensing. Pratihast et al. [27] also consider that data collected by local communities can help validate data from remote sensing, and serve as an additional source of verification.

Data on tree species and forest composition aids accuracy in estimating carbon sequestration. Mingxu Zhao et al. [29]* found that local people can identify tree species nearly as well as trained botanists, but at a much lower cost. Such local skills offer considerable promise for lower cost monitoring, though they may not be available in every community.

### 4. Conveying knowledge and information across governance levels

The main feature of PMRV is the inclusion of local people in contributing to the data flow used in a national MRV system, and–conversely–in benefiting from feedback from the national system on the state of the forests they manage. Information needs to be conveyed across multiple levels of governance. Vega Praputra et al. [22] explored data flow in the Indonesian forestry sector, and found that locally collected forestry data (e.g. yields and diseases of smallholder forest crops, forest disturbances, logging activities) could be merged with the national database in a consistent manner. For this to happen, they note the need for facilitators to be present at the local level (e.g., civil society organizations, government agencies, trained villagers). These facilitators should understand the national MRV system, possess the skills to aggregate data at the village level (e.g. using appropriate software), and ensure data accuracy, completeness, consistency and comparability. McCall et al. [30]* found that, in Mexico, community members with appropriate training and a balance of technical skills and experience can competently collect data needed for MRV. In the Indonesian healthcare system, villagers have for decades been taking measurements and reporting to the sub-district government health center, eventually reaching the national level [23]. Data collected by villagers are considered accurate and are used by the national government to guide planning. As explained earlier, Pratihast et al. [27] proposed a simple web-based system to integrate locally collected data into the national database in near real-time.

### 5. Clarifying and enabling the links to REDD+ safeguards

REDD+ has adopted a set of social and environmental safeguards to ensure that its activities do not result in negative social and environmental impacts. The following principles were agreed during the 2010 Conference of the Parties (COP) in Cancun: transparency, participation of stakeholders, protection of biodiversity and ecosystem services, and respect for the rights of indigenous and local communities [31]. Several studies have found that local people’s participation in MRV can contribute in checking if REDD+ safeguards have been implemented. PMRV can be used to collect information on synergies and tradeoffs between REDD+, social and economic wellbeing, and environmental integrity that would otherwise be difficult to attain.

Bong et al. [20] suggest that communities are best placed to determine promising options to address trade-offs between livelihoods and deforestation and forest degradation. According to McCall et al. [30], local communities have experience and competence in observing and checking (i.e. monitoring) the status of their natural resources, such as water and forest quality, valued wildlife, and territorial infringements. Their interest in this monitoring is often linked to the perceived opportunities for ecotourism. Mingxu Zhao et al., [29], support these observations and suggest that community-led collection of tree diversity data can contribute to monitoring biodiversity safeguards in REDD+ programs.

## Is PMRV feasible?

The studies in this collection suggest that PMRV is feasible and that adopting PMRV can provide opportunities for carbon and non-carbon monitoring to support REDD+ implementation in other ways too, such as:

1. Monitoring REDD+ safeguards
2. Monitoring co-benefits and benefit sharing of REDD+ interventions on the ground
3. Obtaining more accurate information on drivers of deforestation
4. Using multiple approaches for more robust information/verification systems

However, the studies in this collection also indicate several caveats:

1. A feasibility study should be conducted prior to implementing PMRV to check the capacity and possible level of community involvement. Methods of engaging with local communities in MRV will need to be as diverse as the communities involved.
2. Incentives for maintaining local participation need to be developed and should incorporate important non-financial incentives and equity. For example, (i) plan the measurement activities with local people to ensure relevance to the local context (e.g. tenure arrangements, level of ecological knowledge, livelihood activities); and (ii) acknowledge the community’s role and rights as forest managers and beneficiaries, and provide new job and livelihood opportunities for participants.
3. To be ‘PMRV’ ready, national governments need to invest in a reporting and verification system and a national database designed to integrate locally collected data. This should include: (i) building the capacity of facilitators, (ii) simplifying and clarifying reporting procedures across multiple levels of governance; (iii) providing mechanisms to clarify monitoring and reporting responsibilities, and benefits from PMRV, which are often related to local land rights and livelihoods; (iv) using a tiered system that accommodates different levels of reporting capacity across communities and countries involved in REDD+ to progressively achieve the gold standards; and (v) developing PMRV standards and step-by-step guidelines to be included in GOFC-GOLD methods and procedures [7].

In circumstances where local community tenure is unclear and funding and local motivation cannot be secured, a PMRV approach is unlikely to be viable–at least in the short-term.

## Next steps for PMRV research

Currently, PMRV is not widely practiced, however, some governments, including Indonesia, are considering including it in their national MRV systems [32]. To assist in the building of such a system, we need to:

1. **Assess past experiences systematically**: PMRV pilot activities provide lessons on how to sustain local participation and highlight specific challenges. We need to identify how best to evaluate outcomes and lessons.
2. **Assess costs and benefits at multiple levels**: so far, the costs and benefits of PMRV have only been studied at the local level, e.g. comparing the cost of measuring trees with local people versus experts. We need to explore and determine the costs and benefits of scaling up PMRV at regional and national levels, and include all investments required to implement PMRV.
3. **Operationalize reporting**: practical tools are required to facilitate reporting from local to national levels. While we possess a good understanding of how local communities could be involved in data collection, and national databases are already operational in many countries implementing REDD+, we lack agreed formats for data collection and reporting, procedures for aggregating locally collected date and ensuring its inclusion in the national database.
4. **Operationalize verification**: practical approaches are required to facilitate verification. PMRV can be one among multiple approaches, together offering more reliable information than any one approach alone. Key to improved reliability is the recognition of discrepancies between data sets from different sources and effective actions to address them. Both remain unclear so far. While we are certain that PMRV can benefit from local communities' cross checking and monitoring, we need to develop the procedures to ensure that these benefits are forthcoming.
5. **Move from a project scale to a national scale**: developing a larger scale system for PMRV in REDD+ poses challenges. However, we can utilize long term experiences such as from the Indonesian healthcare monitoring system to learn how to build a viable, national, data collection and reporting system.
6. **Test and refine PMRV**: any PMRV implementation needs to build in learning and adaptation procedures. The pilot projects with REDD+ implementation are small scale and PMRV is not fully operational. A large-scale test of PMRV that includes collecting, reporting, and validating data, then integrating the data into a national database, and finally feedback would provide further lessons to guide these important developments.
